# Bioproduction of Quercetin and Rutinose Catalyzed by Rutinosidase: Novel Concept of “Solid State Biocatalysis”

**DOI:** 10.3390/ijms20051112

**Published:** 2019-03-05

**Authors:** Jana Kapešová, Lucie Petrásková, Kristína Markošová, Martin Rebroš, Michael Kotik, Pavla Bojarová, Vladimír Křen

**Affiliations:** 1 Institute of Microbiology of the Czech Academy of Sciences, Laboratory of Biotransformation, Vídeňská 1083, CZ 14220 Prague 4, Czech Republic; janakapesova@gmail.com (J.K.); petraskova@biomed.cas.cz (L.P.); kotik@biomed.cas.cz (M.K.); bojarova@biomed.cas.cz (P.B.); 2 Institute of Biotechnology, Slovak University of Technology, Radlinského 9, 81237 Bratislava, Slovakia; kristina.markosova@stuba.sk (K.M.); martin.rebros@stuba.sk (M.R.)

**Keywords:** *Aspergillus niger*, quercetin, rutin, rutinose, rutinosidase, “solid-state biocatalysis”

## Abstract

Quercetin is a flavonoid largely employed as a phytochemical remedy and a food or dietary supplement. We present here a novel biocatalytic methodology for the preparation of quercetin from plant-derived rutin, with both substrate and product being in mostly an undissolved state during biotransformation. This “solid-state” enzymatic conversion uses a crude enzyme preparation of recombinant rutinosidase from *Aspergillus niger* yielding quercetin, which precipitates from virtually insoluble rutin. The process is easily scalable and exhibits an extremely high space-time yield. The procedure has been shown to be robust and was successfully tested with rutin concentrations of up to 300 g/L (ca 0.5 M) at various scales. Using this procedure, pure quercetin is easily obtained by mere filtration of the reaction mixture, followed by washing and drying of the filter cake. Neither co-solvents nor toxic chemicals are used, thus the process can be considered environmentally friendly and the product of “bio-quality.” Moreover, rare disaccharide rutinose is obtained from the filtrate at a preparatory scale as a valuable side product. These results demonstrate for the first time the efficiency of the “Solid-State-Catalysis” concept, which is applicable virtually for any biotransformation involving substrates and products of low water solubility.

## 1. Introduction

Flavonoids, which occur in plants, vegetables and fruits, are generally considered as efficient antioxidants and chemoprotectants. They are a plentiful but a structurally homogeneous group of plant natural products, whose structure is derived from phenylchromene hydroxylated or methoxylated at various positions. A number of flavonoids are present in natural sources in the form of glycosides. The flavonol quercetin is one of the most common and abundant flavonoids in the plant kingdom. It is a prominent bioactive flavonol in the human diet and its daily intake has increased considerably due to its use as a food supplement [1] and mainly due to the general medical recommendation to “eat five servings of fruits and vegetables a day” [2].

**Figure 1:**
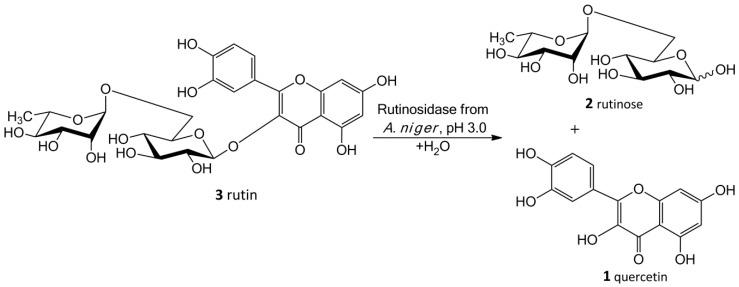
Bioconversion of rutin to quercetin using rutinosidase.

Flavonol quercetin (3,2-(3,4-dihydroxyphenyl)-3,5,7-trihydroxy-4*H*-1-benzopyran-4-one; **1**) frequently occurs in the human diet (typically in apples, onions, buckwheat etc.) in the form of glycosides—the most prominent being quercetin-3-*O*-β-d-glucopyranoside (isoquercitrin) [3] and quercetin-3-*O*-rutinoside (rutin; **3**) (Figure 1) [4]. They have a broad range of positive effects in humans [5] and they are used in a number of medicines and over-the-counter preparations and often in food supplements. Quercetin is largely utilized as a phytochemical remedy for a variety of diseases [5], such as diabetes/obesity and circulatory dysfunctions, inflammation and mood disorders or as an adaptogen. Furthermore, it may be used as an efficient and affordable radioprotectant (e.g., for a large population in case of attack with a “dirty bomb”—radiological dispersal device that combines radioactive material with conventional explosives) [6]. Quercetin used to be considered potentially harmful, since it gave a positive reaction in the Ames’ test, which implied its mutagenicity and potential cancerogenicity [7]. Nevertheless, these doubts have recently been dispelled and the Federal Drug Agency in the US issued GRAS notice for pure quercetin [8]. This certification led to the recent commercial boom in quercetin marketing.

The structure of quercetin is quite unique in terms of antioxidant and antiradical activity: the catechol moiety together with the free OH group at C-3 in conjugation with the C-4 oxo group ensures electron delocalization on ring B. Furthermore, the configuration of OH groups at C-3, -5 and -7 together with the C-4 oxo group make quercetin one of the most potent antioxidants ever discovered [5]. The free C-3 OH group, often glycosylated in natural quercetin glycosides (e.g., rutin, quercitrin, isoquercitrin), is crucial for the high antioxidant activity of quercetin [9,10]. This is the reason why free quercetin has a considerably higher antioxidant activity than its glycosides.

The direct extraction of quercetin from natural sources is impractical, mostly due to the complexity of the matrix and its occurrence in various (glycosylated) forms. An alternative method described in the literature is the oxidation of taxifolin (dihydroquercetin), obtained from Siberian woods, with NaNO_2_ [11]. This multistep method does not yield high-purity quercetin and uses relatively toxic chemicals.

A common starting material for quercetin production is rutin (**3**)—a commodity chemical obtained from various plant materials by extraction (typically from the Brazilian tree Fava d’anta, *Dimorphandra mollis*). Quercetin is currently prepared by acid hydrolysis of rutin, for example, by boiling with diluted HCl [12,13]. This process yields quercetin and the monosaccharides d-glucose and l-rhamnose because rutinose bound to quercetin via the β-glycosidic linkage is completely hydrolyzed. Boiling in a strong acid may lead to a partial decomposition of all reactants, which deteriorates the purity and quality of the final product. Such a product can also hardly be declared to be of “bio” quality.

There are also enzymatic methods for producing quercetin from rutin [13]. These methods typically employ mixtures of enzymes (e.g., β-d-glucosidases and α-l-rhamnosidases) that yield mixtures of the respective monosaccharides and quercetin. Since rutin has a relatively low water solubility (5 g/L at pH 5; 15 g/L at pH 8) and all enzymatic methods described so far require a completely dissolved rutin, they employ cosolvents such as MeOH, EtOH or DMSO [14,15]. The use of cosolvents complicates the procedure as they are flammable, toxic and their residues may contaminate the final product (especially DMSO, which is not easy to remove by evaporation). Moreover, co-solvents often inactivate the enzymes employed and thus decrease the final yields and extend reaction times. Therefore, their use must always be a compromise between the increase in rutin solubility and enzyme denaturation. The low rutin solubility also limits the possibilities of enzyme recycling or the use of immobilized enzymes-cf. [16].

Our new biosynthetic method allows working with rutin in highly concentrated aqueous suspensions (up to ca 300 g/L, ca 0.5 M) without any co-solvents. This method directly yields a precipitated product (**1**) in a virtually quantitative yield that can easily be filtrated and purified just by washing at the filter. The filtrate contains a still active enzyme, which can be reused despite the presence of rutinose from the first reaction—rutinose has no inhibitory activity on the enzyme. The rare and so far unexplored sugar rutinose (**2**) is produced as a side product and can be crystallized from the concentrated filtrate in a high purity (Figure 1). This disaccharide has a potential application in cosmetics. We present here a novel concept of “Solid State Biocatalysis” that enables an exceptionally high space-time-yield.

## 2. Results

### 2.1. Production of Rutinosidase

#### 2.1.1. Production of the Wild-Type Rutinosidase from *Aspergillus niger*

The production and purification of the wild-type rutinosidase from *A. niger* have been previously described in detail [17]. In the scope of this work, the use of the crude, wild-type enzyme for the production of quercetin and rutinose has been tested. Besides the production of rutinosidase (day 6; rutinosidase activity 0.31 U/mL), the co-production of α-l-rhamnosidase (0.30 U/mL) and β-d-glucosidase (0.49 U/mL) during fermentation were observed. This made the use of the crude enzyme unfeasible, mainly due to the parallel cleavage of rutinose and other unwanted reactions. Moreover, the crude wild-type enzyme is not stable (probably due to the presence of proteases in the medium), which was also demonstrated in the tests with rutin bioconversion (see Section 2.2.1). In summary, without enzyme purification, which is not feasible economically at a large scale, the crude wild-type enzyme cannot be used for this process.

#### 2.1.2. Production of Recombinant Rutinosidase from *Aspergillus niger* in *Pichia pastoris*

**Figure 2:**
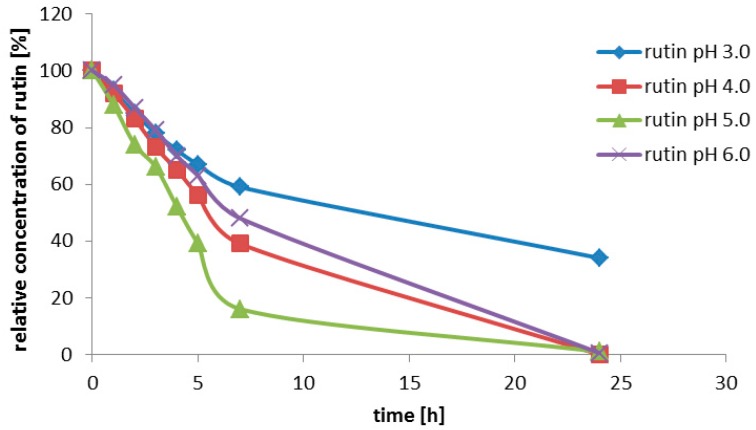
Bioconversion of rutin to quercetin using crude recombinant rutinosidase produced at various pH (rutin concentration 100 g/L; reaction volume 10 mL; 40 °C; pH 3); rutinosidase was produced in the media with the starting pH set to 3–6.

After cloning procedure we observed diminished stability of crude recombinant rutinosidase below pH 3.5 in the cultivation media that were acidified during cultivation in flasks. This instability was obviously caused by the production of (unspecified) serine protease(s), because addition of the protease inhibitor phenylmethanesulfonylfluoride (PMSF, 1 mM) fully restored enzyme stability [18]. The stability of the enzyme was evaluated in the deglycosylation reaction of rutin (100 g/L). We tested flask production in the BMMH medium with the pH set to values of 3.0; 4.0; 5.0; 6.0. Rutinosidase activity in the medium was 0.080; 0.056; 0.062; and 0.046 U/mL, respectively. Although the specific enzyme production was the highest in the most acidic medium (pH 3.0), the performance of the enzyme produced under these conditions was the worst since the conversion was not completed even after 24 h. The highest initial activity and the highest stability were found for the enzyme produced at pH 5.0 (Figure 2). This figure shows the fastest turnover of the substrate with enzyme obtained by the cultivation at pH 5.0 (green curve), which is the most stable. Enzyme obtained from the medium of pH 3.5 has a high activity but its overall performance is afflicted by its lower stability. This also demonstrates the importance of pH control of the fermentation for enzyme stability.

#### 2.1.3. Scale-Up of the Production of Recombinant Rutinosidase

**Figure 3:**
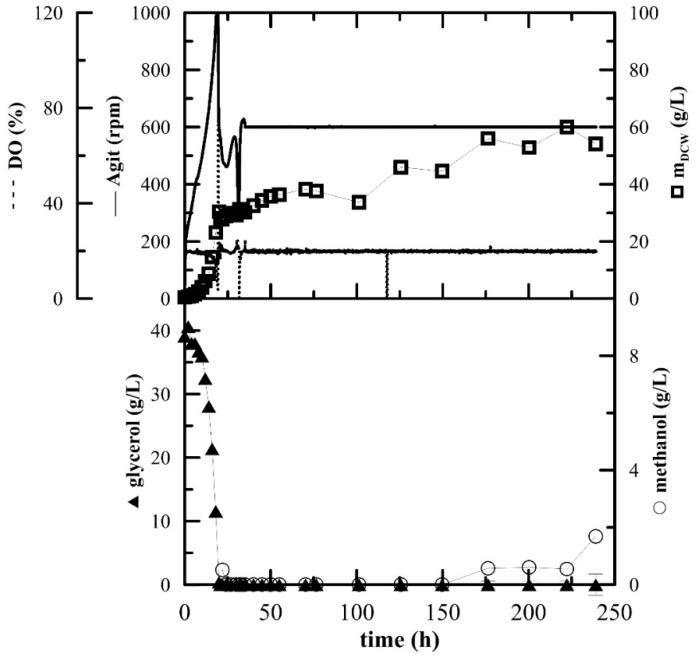
Fed-batch cultivation of *P. pastoris* expressing rutinosidase with methanol feeding according to the actual level of dissolved oxygen. Conditions: 1.5 L BSM, 5% inoculum, 30 °C, pH 5.0, DO 20%, stirring cascade 50–1000 rpm; after 35 h fixed at 600 rpm.

The production of recombinant rutinosidase by *P. pastoris* KM71H was scaled up in 3-L laboratory bioreactors. Glycerol was depleted after approximately 20 h, during which the exponential growth of biomass was observed (Figure 3). Then, two methanol pulses of 3 g/L were added at hour 21 and hour 32. Fed-batch methanol feeding was started at hour 35, according to the optimized protocol described by [19]. During the fed-batch phase, methanol was fed according to the actual level of dissolved oxygen and never exceeded a concentration of 3 g/L, which is toxic for this *P. pastoris* Mut^S^ strain. The concentration of methanol started to increase slightly at the end of the fermentation and also the growth of biomass steadied, which indicated that the biomass reached the stationary phase and the fermentation was terminated. SDS-PAGE electrophoresis (Figure S1, Supplementary Material) showed a band of approx. 66 kDa, representing the produced rutinosidase. The absence of other significant protein bands demonstrates the great advantage of controlled fermentation, which produces no other extracellular proteins than the desired rutinosidase. The overall productivity of the produced rutinosidase was 5.69 mg_prot_·L^−1^·h^−1^.

### 2.2. Bioconversion of Rutin to Quercetin

#### 2.2.1. Bioconversion of Rutin to Quercetin Using Wild-Type Rutinosidase

The first tests of a larger-scale conversion of rutin to quercetin were performed with the wild-type enzyme. The reaction was successfully accomplished at analytical scale with the purified wild-type rutinosidase (up to 100 g/L rutin). However, for a practical large-scale biotechnological application protein purification is not economically feasible. Therefore we also tested crude rutinosidase (centrifuged medium from the cultivation of *A. niger*, adjusted to pH 3 with phosphoric acid, diluted twice with water). The rutin concentrations were 50, 100, 150 and 200 g/L. Only the reaction with 50 g/L was completed within 24 h whereas higher concentrations of rutin led to incomplete conversion (Figure S2, Supplementary Material). It seems that the enzyme is partially inactivated during the reaction with a higher rutin concentration, possibly by some proteases in the crude medium. Moreover, the crude medium also contains a considerable activity of α-l-rhamnosidase (rutinosidase 0.305 U/mL, α-l-rhamnosidase 0.30 U/mL), which causes degradation of rutinose—valuable by-product of this bioconversion.

#### 2.2.2. Bioconversion of Rutin to Quercetin Using Recombinant Rutinosidase

##### Optimization of Reaction Conditions

The recombinant rutinosidase from *A. niger* produced in *P. pastoris* has a temperature optimum of 50 °C at pH 3.5; temperatures exceeding 55 °C resulted in a loss of activity [17]. The stability of the recombinant (crude, dialyzed) enzyme at different pH and temperatures was tested. The enzyme loses virtually all activity in ca 2 h at 50 °C. (Figure S3a, Supplementary Data). Therefore a lower temperature (40 °C), where the enzyme has an activity of ca 60% compared to the optimum temperature but is quite stable, was selected.

Nevertheless, even at 50 °C the performance of rutinosidase during rutin bioconversion was very satisfactory. The complete conversion of rutin at a concentration of 200 g/L was accomplished within 5–6 h (Figure S3b, Supplementary Material). It is obvious that the enzyme in the presence of substrate is much more stable than in a mere buffer.

**Figure 4:**
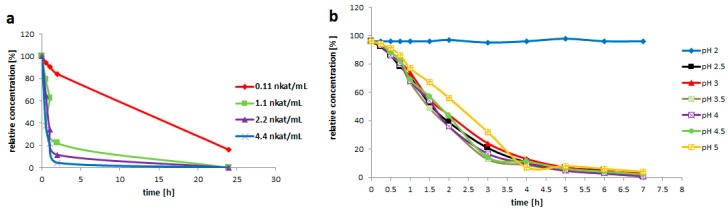
Optimization of reaction conditions for bioconversion of rutin to quercetin. (**a**) Bioconversion of rutin using various amounts of pure rutinosidase (rutin 200 g/L; reaction volume 3 mL; 35 °C, pH 3.0). (**b**) Bioconversion of rutin in 0.2 M glycine buffer at various pH (rutin 200 g/L, reaction volume 3 mL, 35 °C, purified rutinosidase 0.2 U/mL).

The pH optimum of recombinant rutinosidase is at pH 3.0. At pH 5.0 the enzyme still maintains 50% of its maximum activity and at pH 2.0 and pH 7.0 it is virtually inactive [17]. To support the above hypothesis the performance of the enzyme in a real reaction system with a high rutin concentration (200 g/L) was tested at different pH. The results showed (Figure 4b) that at pH 2.0 the enzyme is inactive and its performance is suboptimal at pH 5.0. However, from pH 2.5 to 4.5 the enzyme performance is—in this experimental setup—practically the same. Therefore, for further experiments pH 3.0 was selected as the optimum one for rutin bioconversion.

Furthermore, the activity of enzyme in the reaction system containing 200 g rutin/L was optimized. The critical rutinosidase activity seems to be ca 0.1 U/mL. At a rutinosidase activity of 0.2 U/mL, the reaction was completed within ca 6 h (Figure 4a).

##### Enzyme Storage and Stability

For the purposes of potential biotechnological applications, it is vital to assess the possibilities of long-term storage of the enzyme. The enzyme (both wild type and purified recombinant) is stable for several months at 6 °C. Moreover, we found that recombinant rutinosidase may be lyophilized from the crude medium without any notable loss of catalytic activity. The rutinosidase activity of the lyophilized sample resuspended in 50 mM citrate-phosphate buffer pH 3.0 was at least 90% compared to the fresh enzyme with *p*NP-rutinoside. The comparison of rutin bioconversion reactions with the lyophilized enzyme after shock or slow freezing (Figure S4a,b Supplementary Material) shows that shock freezing is a preferable method due to its mildness and full preservation of rutinosidase activity.

##### Optimization of the Substrate Concentration and Scale-Up

**Figure 5:**
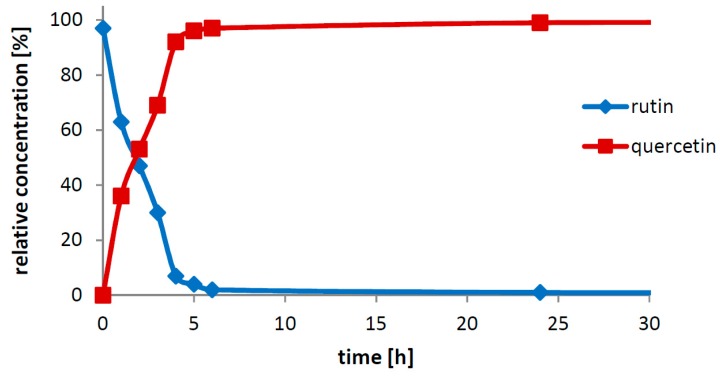
Bioconversion of rutin to quercetin under optimum conditions. Rutin 200 g/L; reaction volume 30 mL; 40 °C; pH 3.0; crude recombinant rutinosidase activity 0.8 U/mL.

The substrate concentration is crucial to achieve the optimal space-time-yield. The substrate concentrations considerably higher than 100 g/L, for example, 200, 300, 450 g/L rutin in reaction volumes of 30 and 100 mL and also a concentration of 450 g/L in a volume of 500 mL were tested. The crude recombinant enzyme prepared in the fermenter under controlled pH (0.8 U/mL) gave 100% conversion in all reaction setups within 24 h. For practical applications, 200 g/L (Figure 5) seems to be the highest suitable substrate concentration due to the fact that at very high rutin concentrations the reaction mixture tends to become significantly more viscous (especially after reaction completion) and it needs to be diluted with water for future processing.

Reaction scale-up was tested with a substrate concentration of 200 g/L in volumes of 50, 100, 250 mL and 2 L (Figure S5, Supplementary Material) using as little as 0.2 U/mL rutinosidase. In all the reaction set-ups, the conversion was completed within ca 6 h yielding quercetin in virtually quantitative yield.

### 2.3. The Concept of “Solid-State Biocatalysis”

**Figure 6:**
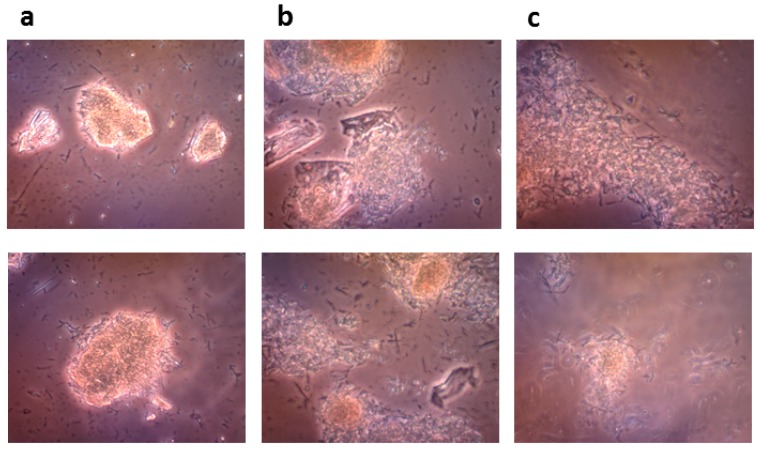
Microscopic monitoring of “Solid-State Biocatalysis” after (**a**) 0 h, (**b**), 2 h and (**c**), 5 h of reaction (rutin 100 g/L, crude recombinant rutinosidase). OLYMPUS CX41 optical microscope equipped with OLYMPUS U-CMAD3 digital camera, magnification 400×.

Since the bioconversion of rutin into quercetin and rutinose proceeds in a very thick suspension, we were interested to see how the reaction runs at the microscopic level. The monitoring of the reaction mixture with an optical microscope (Figure 6) showed that the starting material was indeed present in crystalline form and over the course of the reaction (2 and 5 h) multiple small crystals of the product (most probably quercetin) were formed, which gradually substituted the large crystals of rutin as the reaction proceeded (Figure 6c).

## 3. Discussion

The above results clearly demonstrate that the rutinosidase from *Aspergillus niger* is a highly attractive enzyme for biotechnological application. Its heterologous expression elegantly solved the problem of contaminating α-l-rhamnosidase and β-d-glucosidase activities co-produced with the wild-type enzyme, and, at the same time, allowed a multiple-litre production scale-up, which can be further upscaled if convenient. Moreover, due to the production selectivity, the robust crude medium can directly be applied for a one-step bioproduction of a GRAS-classified phytochemical quercetin with a high space-time yield. Here, we must highlight the necessity of a thorough search for the optimum reaction conditions. As previously confirmed with other glycosidases, the temperature optimum may not copy the temperature stability [20]. Admittedly, the mere activity measurement profiles under given conditions do not always reflect the real situation during the biocatalytical process. There, the enzyme active site is protected against the detrimental effect of extreme conditions such as high temperature by the proceeding biocatalytical reaction. This was clearly visible comparing the results of stability measurements and the real reaction outcome at 50 °C (Section 2.2.2). Interestingly, this effect is observed even though no inhibition by the released hydrolytic product (rutinose) occurred. Rutinosidase inhibition with rutinose has been tested and up to 0.5 M rutinose (corresponding to ca 300 g/L starting concentration of rutin) the rutinosidase activity remains unchanged (data not shown). Apparently, since the enzyme active site is constantly occupied by the incoming reaction substrate in excess, its architecture is well protected against the impact of detrimental conditions. Thus, the enzyme is obviously more stable in a bioconversion reaction with a high rutin concentration than the enzyme incubated under the same conditions in a mere buffer.

The crucial finding presented in this work is the particular biocatalytic set-up that we denoted as “Solid-State-Biocatalysis.” From the above data, it is obvious that the bioconversion of rutin into quercetin and rutinose catalyzed by rutinosidase proceeds in a very thick suspension (tested up to 450 g/L ≈ 0.7 M), where both the starting material and product remain primarily in the solid phase (the typical solubility of both reactants is ca 1–3 g/L, with quercetin being less soluble than rutin). Numerous authors tested this and other similar reactions, typically the partial hydrolysis of rutin into isoquercitrin with α-l-rhamnosidase, with the addition of various cosolvents [21]. In these reaction set-ups, authors barely achieved concentrations of dissolved rutin higher than approx. 10 g/L, following the commonly accepted scheme that enzymatic reactions proceed solely or at least in majority in the liquid phase. In the present case, in contrast, the majority of substrate is present in the solid phase. We suppose that the reaction proceeds in the (over)saturated microenvironment solution of the substrate crystals. The formed product, also badly water soluble, precipitates quickly out of this microenvironment, thereby constantly shifting the reaction equilibrium to the full conversion.

There are obviously two major driving forces of the reaction: (i) the high affinity of the biocatalyst for the substrate, enabling its conversion at a low concentration and (ii) the thermodynamic shift of equilibrium towards product formation caused by continuous product “removal” due to its precipitation. This type of reaction apparently runs well when both reactants are poorly soluble and the catalyst is efficient. Interestingly, when a cosolvent (e.g., 5% DMSO) is added into the reaction mixture, the reaction is slower and the conversion is not complete. This corroborates the above hypothesis because with the addition of the cosolvent, the product occurs in higher concentrations (inhibition with a higher concentration of quercetin) in the reaction mixture due to its increased solubility. A partial inactivation of the enzyme by the cosolvent cannot be excluded either.

Another advantage of the “Solid-State Biocatalysis” setup is the resistance of rutinosidase towards product inhibition by the disaccharide rutinose. On the contrary, the accumulation of rutinose in the reaction mixture and the running catalytic reaction stabilize the enzyme, as observed in our stability studies (see Section 2.2.2 and Figure S3, Supplementary Material). Rutinose in a higher concentration may also diminish the solubility of quercetin, lowering thus its concentration.

The main product of this enzymatic process is pure quercetin that does not involve any harmful and irritant chemicals, which makes it applicable in the cosmetics and food industries as a “Bio-quality” product. This procedure also yields the valuable side product rutinose, which has not been available in multigram/kilogram scale for a reasonable price so far. Rutinose can be obtained from the filtrate of the reaction mixture after short boiling with charcoal, calcium hydroxide and Celite (each 0.5 g/L), which removes protein and phosphate impurities and decolorizes the solution. After filtration and evaporation, pure rutinose (>97%) is obtained by crystallization or lyophilization. This opens possibilities for research and potential application of this disaccharide for example, in cosmetic applications because α-l-rhamnose and/or α-l-rhamnosides were found to interact with specific receptors on keratinocytes, which play an important role in cell and (skin) tissue aging [22].

## 4. Materials and Methods

### 4.1. Materials

The enzymes and buffers for DNA manipulations were obtained from New England Biolabs (Ipswich, MA, USA). Media components were from Oxoid (Basingstoke, UK) or Carl-ROTH (Karlsruhe, Germany). Rutin was from Alchimica (Prague, Czech Republic) and other chemicals were purchased from Sigma-Aldrich.

### 4.2. Biological Material

An EasySelect *Pichia pastoris* KM71H Expression Kit was purchased from Invitrogen (Waltham, MA, USA). This expression system employs the AOX1 (alcohol oxidase 1) promoter, inducible by methanol.

The culture of *Aspergillus niger* K2 CCIM is deposited in the Collection of Microorganisms of the Institute of Microbiology of the Czech Academy of Sciences, Prague. The culture is maintained on slants [g/L]: agar-agar, 20; bacto-peptone, 5; and malt extract, 35.

### 4.3. Media

The inoculum for *P. pastoris* cultivation was prepared in BMGY medium (Buffered Glycerol-Complex Medium) [g/L]: yeast extract, 10; peptone, 20; 100 mM potassium phosphate, pH 6.0; Yeast Nitrogen Base (YNB, Oxoid cz., Brno, Czech Republic), 13.4; biotin 0.0004; glycerol, 10.

Buffered Methanol-Complex Medium (BMMY) has the same composition as BMGY but instead of 1% (*w*/*v*) glycerol methanol (0.5%, *v*/*v*) is added.

For large-scale productions, minimal media were used. BMGH medium (Buffered Minimal Glycerol Medium) [g/L]: 100 mM potassium phosphate pH 6.0; YNB, 13.4; biotin 0.0004; glycerol, 10 for overnight preculture and BMMH medium (Buffered Minimal Methanol Medium) [g/L]: 100 mM potassium phosphate pH 6.0; YNB, 13.4; biotin 0.0004; methanol, 5).

Fed-batch fermentations were carried out in BSM medium (Basal Salt Medium) [g/L]: 85% H_3_PO_4_, 26.7 mL; CaSO_4_·2H_2_O, 1.17; K_2_SO_4_, 18.2; MgSO_4_·7H_2_O, 14.9; KOH, 4.13; and glycerol, 40; and supplemented with 4.35 mL/L of PTM_1_ (trace salts solution) [g/L]: CuSO_4_·5H_2_O, 6; NaI, 0.08; MnSO_4_·H_2_O, 3; Na_2_MoO_4_·2H_2_O, 0.2; H_3_BO_3_, 0.02; CoCl_2_, 0.5; ZnCl_2_, 20; FeSO_4_·7H_2_O, 65; biotin, 0.2; H_2_SO_4_ conc. 9.2 g). Methanol added in fed-batch experiments was also supplemented with PTM_1_ (1.2 mL/L pure methanol).

The production medium for *A. niger* cultivation of consisted of [g/L]: rutin, 5.0; KH_2_PO_4_, 15.0; NH_4_Cl, 4.0; KCl, 0.5; yeast extract, 5.0; casein hydrolysate, 1.0; and 1.0 mL of trace element Vishniac solution [23] at pH 5.0. The pH of the medium was adjusted to 5.0. After sterilization, each flask was supplemented with 1.0 mL of sterile 10% MgSO_4_·7H_2_O (*w*/*v*).

### 4.4. Preparation of Enzymes

#### 4.4.1. Cultivation of *Aspergillus niger*

The production medium (200 mL) was inoculated with a suspension of spores in a 0.1% (*v*/*v*) Tween 80 solution and cultivated in 500-mL Erlenmeyer flasks on a rotary shaker at 28 °C and 250 rpm. The mycelium of *A. niger* K2 CCIM was cultivated for 12 days at 28 °C and 250 rpm in Erlenmeyer flasks in the presence of 0.5% *w*/*v* rutin as the rutinosidase inducer. The mycelium was subsequently removed by filtration through an asbestos-cellulose filter (C10, Vertex CZ, Prague, Czech Republic). The filtrate was directly used (as a crude wild rutinosidase) for rutin conversion.

#### 4.4.2. Heterologous Expression of Rutinosidase in *Pichia pastoris*

The expression vector pPICZαA-RUT, obtained as described previously [17] was linearized with restriction endonuclease *Sac*I and electroporated to competent *P. pastoris* cells according to the manufacturer’s instructions (EasySelect *Pichia* Expression Kit, Invitrogen; Waltham, MA, USA). The electroporated cells were grown at various concentrations of zeocin (100 µg/mL) on YPD (Yeast Extract Peptone Dextrose Medium; yeast extract OXOID 1%, bacteriological peptone OXOID 2%, glucose 2% (LACHNER, Neratovice, Czech Republic)) agar plates for 2 days at 28 °C.

The production of recombinant rutinosidase was performed according to the manufacturer’s instructions as follows: the colonies were inoculated into 100 mL of BMGY medium pH 6.0 and incubated overnight with shaking at 28 °C. The cells were collected by centrifugation (5000× *g*, 10 min, 20 °C) and the pellet was resuspended in 30 mL of BMMY medium (see Section 4.3 Media) in a 300 mL baffled flask. The production of rutinosidase was induced by the addition of methanol (0.5% *v*/*v*) every 24 h for 4 days. The flasks were incubated at 28 °C and 220 rpm.

For the large-scale production of rutinosidase, we used 1 L of BMGH medium for overnight preculture. The cells were then collected and resuspended in 200 mL of BMMH medium and incubated at 28 °C on a rotary shaker with methanol induction (0.5% *v*/*v*) every 24 h for 4 days.

#### 4.4.3. Fermentation Scale-Up

##### Fermentation Media

The inoculum was prepared in BMGY medium. Fed-batch fermentations were carried out in BSM medium; methanol added in fed-batch experiments was also supplemented with PTM_1_ (1.2 mL/L methanol).

##### Bioreactor Cultivation

The inoculum for the fermentation cultivation was prepared in 100 mL of BMGY medium. The fermentation was essentially performed as described previously [20] in 3-L laboratory fermenters (Brunswick BioFlo^®^ 115, Eppendorf, Hamburg, Germany). Then, 1.5 L of BSM media supplemented with 6.53 mL of PTM_1_ was inoculated with inoculum (OD_600_ approx. 10–12) to a concentration of 5% *v*/*v*. The fermentation conditions were as follows: 30 °C, pH 5 maintained with ammonium solution (28–30%), DO (dissolved oxygen) 20% maintained by agitation cascade from 50 to 1000 rpm, aeration 0.66 vvm with the addition of 200 µL of Struktol J650 (Schill + Seilacher “Struktol” GmbH, Hamburg, Germany) as an antifoaming agent. After the complete depletion of glycerol (approx. 20 h), two methanol pulses of 3 g/L were added after 20 and 31 h. After the depletion of the second pulse, the agitation was fixed at 600 rpm, the agitation cascade was stopped and additional methanol (3 g/L) was added. The methanol feed was connected to the actual level of dissolved oxygen as described in Reference [20]. Whenever the level of DO rose above 20%, the methanol feed was turned on by an automated program and when the DO level rose above 30%, signalling an excess of methanol and the inability of the culture to utilize it, the pump was turned off again.

### 4.5. Purification of Recombinant Rutinosidase

Recombinant rutinosidase was purified from the culture medium of *P. pastoris* after 6 days of cultivation with methanol induction. The cells were harvested by centrifugation (5000× *g*, 10 min at 4 °C). The supernatant was dialyzed against 6 L of 10 mM sodium acetate buffer, pH 3.6, for 2 h (dialysis tubing cellulose membrane, Sigma-Aldrich, cut-off 10 kDa). The pH of the solution was then adjusted to 3.6 with 10% acetic acid and filtered. This solution was loaded into a Fractogel EMD SO_3_^−^ column (15 × 100 mm) in 10 mM sodium acetate buffer, pH 3.6. The protein was eluted using a linear gradient of 0–1 M NaCl (5 mL/min). Fractions were collected and then analyzed for rutinosidase activity using *p*-nitrophenol rutinoside as a substrate. The fractions containing rutinosidase activity were concentrated by ultrafiltration using cellulose membranes with a 10 kDa cut-off (Millipore, Merck, Darmstadt, Germany). The concentrated protein was then purified to homogeneity by gel filtration in a Superdex 200 10/300 GL column (10 × 300 mm, 10 mM citrate-phosphate buffer, pH 5.0, 150 mM NaCl).

Protein concentrations were determined by Bradford assay calibrated for bovine serum albumin. The purity of recombinant rutinosidase was checked by 12% SDS-PAGE.

### 4.6. Storage of Enzymes

The purified or crude rutinosidase (as a cultivation medium from transformed *P. pastoris*) could be stored with no notable loss of activity in a fridge (ca 6 °C) for a minimum of 6 months. For lyophilization, the crude medium was rapidly frozen in a liquid nitrogen bath (shock freezing) or frozen in a deep freezer (−80 °C, slow freezing). The material was then lyophilized to dryness. The loss of specific activity of rutinosidase was in both cases under 10%. The lyophilized enzyme can be stored in a fridge in a tightly closed vessel for a minimum of 6 months with no loss of activity.

### 4.7. Enzyme Activity Assay

The rutinosidase activity was measured spectrophotometrically using *p*-nitrophenyl rutinoside as a substrate at a starting concentration of 2 mM. The reaction mixture contained substrate (10 mM solution, 10 µL), 50 mM citrate-phosphate buffer pH 5.0 (10 µL) and the enzyme solution (30 µL). After incubation of the reaction mixture at 35 °C for 10 min with shaking at 850 rpm, 0.1 M Na_2_CO_3_ (1 mL) was added to the reaction mixture. The released *p*-nitrophenol was determined spectrophotometrically at 420 nm. One unit of enzymatic activity was defined as the amount of enzyme releasing 1 µmol of *p*-nitrophenol per minute in 50 mM citrate-phosphate buffer at pH 5.0.

#### Enzymatic Preparation of Colorimetric Substrate *p*-Nitrophenyl Rutinoside

The colorimetric substrate *p*-nitrophenyl rutinoside (not commercially available) was prepared by the glycosylation of *p*-nitrophenyl glucopyranoside using α-l-rhamnosidase (*A. terreus*) and a free rhamnose yielding the title substrate, however with very low yields (ca 3%) [17]. Therefore, we developed another method for the preparation of this compound, taking advantage of the high transglycosylation activity of rutinosidase from *A. niger* towards phenolic substances [17,24]. The following protocol was used: *p*-nitrophenol (100 mg) and rutin (200 mg) were dissolved in DMSO (1.1 mL) and crude rutinosidase (cultivation medium of transformed *P. pastoris*; 6.4 mL, 0.4 U/mL) was added. The pH was adjusted to 3.0 and the mixture was incubated at 40 °C for 24 h. The reaction was monitored by TLC and HPLC. The reaction was stopped by boiling for 10 min, filtered, the pH was adjusted to 7.5–7.7 and unreacted *p*-nitrophenol was removed by extraction (3 × 30 mL EtOAc). The aqueous phase was evaporated, the residue was dissolved in 20% *v*/*v* methanol in water (2 mL) and loaded into a Sephadex LH-20 column (eluted with 20% *v*/*v* methanol). Fractions containing the product (TLC, Silicagel 60 F_254_, Merck; *n*-propanol/H_2_O/NH_4_OH = 7:2:1, *v*/*v*/*v*) were pooled and evaporated to yield 47 mg (32% related to rutin) of the title compound.

### 4.8. Bioconversion of Rutin to Quercetin

#### 4.8.1. Analytical Scale

Purified recombinant rutinosidase from *P. pastoris* was used for the optimization of biotransformation conditions.

##### pH Optimum

Rutin (3, 200 g/L) was suspended in the enzyme solution, which contained 0.2 M glycine-buffer and the pure enzyme (0.2 U/mL). The buffer pH was adjusted to 2.0; 2.5; 3.0; 3.5; 4.0; 4.5 or 5.0. The solution (3 mL) was incubated at 35 °C with shaking (750 rpm) for 7 h. Samples were taken every 30 min for the HPLC analysis.

##### Enzyme Activity

Rutin (3, 200 mg/mL) was suspended in the solution (3 mL) containing 0.2 M glycine-buffer pH 3.5 and the pure enzyme of various activities (0.021; 0.2; 0.4 or 0.8 U/mL). The reaction mixture was incubated at 35 °C with shaking (750 rpm) for 24 h. The reaction was monitored by HPLC. 1 U is defined as the amount of enzyme converting 1 µM of substrate within 1 min under the given conditions.

##### Temperature Optimum

Rutin (3, 200 g/L) was suspended in a solution (10 mL) containing 0.2 M glycine-buffer pH 3.5 and pure enzyme (0.2 U/mL). The reaction mixture was incubated at 10 °C; 20 °C; 35 °C; 40 °C or 50 °C with shaking (750 rpm) for 24 h. Samples were taken every 30 min for the HPLC analysis.

##### Thermostability

The crude medium (*P. pastoris*) containing rutinosidase was adjusted to pH 3.0 and was incubated at 50 °C for 150 min. Every 10 min, samples (30 µL) were taken and the enzyme activity was measured using the above procedure.

#### 4.8.2. Scale-Up of Bioconversion of Rutin to Quercetin

##### Bioconversion Scale-Up Using Crude Wild-Type Rutinosidase (*A. niger*)

For the scale-up process, the centrifuged crude medium from the cultivation of the production strain *A. niger* containing rutinosidase (0.3 U/mL) was used. The crude medium was diluted twice with water and the pH was adjusted to 3.5. The medium itself had a good buffering capacity. Rutin (3; 50 g/L; 100 g/L; 150 g/L or 200 g/L) was suspended in the buffered enzyme solution, incubated at 40 °C with shaking (750 rpm) and the reaction conversion was monitored by HPLC.

##### Bioconversion Scale-Up Using Crude Recombinant Rutinosidase (*P. pastoris*)

For the scale-up process, the crude medium (prepared in a fermenter—see scale up) from transformed *P. pastoris* (containing typically 0.4 U/mL) was adjusted to 0.2 U/mL with water and the pH was adjusted to 3.5 with H_3_PO_4_. The medium itself had a good buffering capacity. Rutin (3; 200 g/L; 300 g/L or 450 g/L) was suspended in the buffered enzyme solution, incubated at 40 °C with shaking (750 rpm) and the reaction conversion was monitored by HPLC. Rutin was completely consumed after 24 h; then the reaction was stopped by heating to 99 °C for 5 min. The reaction mixture was then centrifuged (5000× *g*, 10 min at 20 °C) or filtered.

#### 4.8.3. Microscopic Monitoring of the Solid State Biocatalysis

The progress of bioconversion of rutin to quercetin catalyzed by the crude recombinant rutinosidase (rutin 100 g/L) was monitored with an OLYMPUS CX41 optical microscope equipped with an OLYMPUS U-CMAD3 digital camera (Olympus Europa SE & Co., KG, Hamburg, Germany).

### 4.9. Analytical methods

#### Analytical HPLC

HPLC analyses were performed in a Shimadzu Prominence LC analytical system (Shimadzu, Kyoto, Japan) consisting of the following Shimadzu components: LC-20AD binary HPLC pump, CTO-10AS column oven, SIL-20ACHT cooling autosampler, CBM-20A system controller and SPD-20MA diode array detector. The sample (20 μL) was dissolved in DMSO (150 μL) and analyzed by a Chromolith Performance RP-18e column (100 × 3 mm, Merck, Germany) coupled with an RP-18e guard cartridge kit (5 × 4.6 mm, Merck, Darmstadt, Germany). Binary gradient elution was used: mobile phase A (*v*/*v*): 5% acetonitrile, 0.1% formic acid; mobile phase B (*v*/*v*): 80% acetonitrile, 0.1% formic acid; gradient: 7–25% B for 0–3 min, 30% B for 3–5 min; 7% B for 5–7.5 min. The flow rate was 1.5 mL/min at 25 °C and the injection volume was 0.1 μL; peaks were detected at 360 nm. Retention times [min]: rutin, 2.380; quercetin, 3.964. The authenticity of the compounds, for example, rutin and quercetin, was re-confirmed by MS spectra using LC-MS with commercial samples. Structure of rutinose was confirmed by ^1^H and ^13^C NMR [17].

Glycerol and methanol concentrations were measured by HPLC with an Agilent Technologies 1220 Infinity LC apparatus combined with an Agilent Technologies 1260 Infinity RI detector (Agilent Technologies, Waldbronn, Germany), a WATREX Polymer IEX H form 8 μm, 250 × 8 mm as the main column and a WATREX Polymer IEX H form 8 μm, 40 × 8 mm (WATREX, Prague, Czech Republic) as a guard column, at a flow rate of 0.8 mL/min of 9 mM sulfuric acid at 45 °C.

## 5. Conclusions

We present a novel biotechnological process for the production of the bioactive flavonoid quercetin from the inexpensive starting material rutin. The main feature of the process can be described as “solid-state” enzymatic conversion with both substrate and product being mostly in an undissolved state. The enzymatic reaction itself thus proceeds in the microenvironment solution of the reactants. This new concept of “solid-state biocatalysis” takes advantage of the low water solubility of both the starting material and product. Using this approach with a specific diglycosidase, rutinosidase, quercetin is obtained from the quantitative bioconversion of rutin. Recombinant rutinosidase is used as a crude enzyme directly from a bioreactor cultivation (after biomass centrifugation). In addition, the procedure leads to the formation of the valuable and commercially unavailable disaccharide rutinose as a second product of the reaction. This work demonstrates an elegant approach to biotransformation under mild conditions in the absence of co-solvents and toxic chemicals, potentially transferable to conversions of other natural compounds and food additives. Thanks to the new biocatalytic methodology void of any harmful chemicals, the new process, which results in the products quercetin and rutinose, can be declared as environmentally friendly or a “bio” quality product on the market.

## Supplementary Materials

Supplementary materials can be found at https://www.mdpi.com/1422-0067/20/5/1112/s1.

## Abbreviations

GRAS	Generally-Recognized-As-Safe
DMSO	Dimethylsulfoxide
DCW	Dry Cell Weight
DO	Dissolved Oxygen
BMMY	Buffered Methanol-Complex Medium
BMGY	Buffered Glycerol-Complex Medium
BMGH	Buffered Minimal Glycerol Medium
BMMH	Buffered Minimal Methanol Medium
BSM	Basal Salt Medium
PTM_1_	Trace Salt Solution
YNB	Yeast Nitrogen Base
vvm	volume of air per volume of liquid per minute

## References

[1] Russo M., Spagnuolo C., Tedesco I., Bilotto S., Russo G.L. (2012). The flavonoid quercetin in disease prevention and therapy: Facts and fancies. Biochem. Pharmacol..

[2] Almeida A.F., Borge G.I.A., Piskula M., Tudose A., Tudoreanu L., Valentová K., Williamson G., Santos C.N. (2018). Bioavailability of quercetin in humans with a focus on inter-individual variation. Compr. Rev. Food Sci. Food Saf..

[3] Valentová K., Vrba J., Bancířová M., Ulrichová J., Křen V. (2014). Isoquercitrin: Pharmacology, toxicology, and metabolism. Food Chem. Toxicol..

[4] Gullon B., Lu-Chau T.A., Moreira M.T., Lema J.M., Eibes G. (2017). Rutin: A review on extraction, identification and purification methods, biological activities and approaches to enhance its bioavailability. Trends Food Sci. Technol..

[5] D’Andrea G. (2015). Quercetin: A flavonol with multifaceted therapeutic applications?. Fitoterapia.

[6] Özyurt H., Çevik Ö., Özgen Z., Ozden A.S., Cadirci S., Elmas M.A., Ercan F., Gören M.Z., Şener G. (2014). Quercetin protects radiation-induced DNA damage and apoptosis in kidney and bladder tissues of rats. Free Rad. Res..

[7] Schimmer O., Kruger A., Paulini H., Haefele F. (1994). An evaluation of 55 commercial plant-extracts in the Ames mutagenicity test. Pharmazie.

[8] Andres S., Pevny S., Ziegenhagen R., Bakhiya N., Schäfer B., Hirsch-Ernst K.I., Lampen A. (2017). Safety aspects of the use of quercetin as a dietary supplement. Mol. Nutr. Food Res..

[9] Carullo G., Cappello A.R., Frattaruolo L., Badolato M., Armentano B., Aiello F. (2017). Quercetin and derivatives: Useful tools in inflammation and pain management. Future Med. Chem..

[10] Biler M., Biedermann D., Valentová K., Křen V., Kubala M. (2017). Quercetin and its analogues: Optical and acido-basic properties. Phys. Chem. Chem. Phys..

[11] Levdanskii V.A., Polezhaeva N.I., Kuznetsov B.N. (2001). Method for Producing Quercetin, Involves Oxidizing Larch Wood with Sodium Nitrite and Hydrolysis by Superheated Vapor and Subjecting to Rapid Decompression. Russian Patent.

[12] Zhao Z. (2011). Production of Quercetin by Mixing Inorganic Strong Acid and Water, Adding Rutin, Boiling, Filtering, Hydrolyzing, Washing Cake with Water, Drying, Mixing Cake with Methanol, Filtering, Washing Cake with Methanol and Water, and Drying. Chinese Patent.

[13] Wang J., Zhao L.-L., Sun G.-X., Liang Y., Wu F.-A., Chen Z., Cui S. (2011). A comparison of acidic and enzymatic hydrolysis of rutin. Afr. J. Biotechnol..

[14] Cui X., Wang Z. (2010). New Rutin Hydrolase Used for Preparing Quercetin From Rutin, Prepared by Extracting Tartary Buckwheat Powder Using Acetate Buffer, and Processing Extract in Anion Exchange Chromatography and Gel Chromatography. Chinese Patent.

[15] Nam H.K., Hong S.-H., Shin K.-C., Oh D.-K. (2012). Quercetin production from rutin by a thermostable beta-rutinosidase from *Pyrococcus furiosus*. Biotechnol. Lett..

[16] Rebroš M., Pilniková A., Šimčíková D., Weignerová L., Stloukal R., Křen V., Rosenberg M. (2013). Recombinant α-L-rhamnosidase of *Aspergillus terreus* immobilization in polyvinylalcohol hydrogel and its application in rutin derhamnosylation. Biocatal. Biotransform..

[17] Šimčíková M., Kotik M., Weignerová L., Halada P., Pelantová H., Adamcová K., Křen V. (2015). α-L-Rhamnosyl-β-D-glucosidase (rutinosidase) from *Aspergillus niger*: Characterization and synthetic potential of a novel diglycosidase. Adv. Synth. Catal..

[18] Salamin K., Sriranganadane D., Léchenne B., Jousson O., Monod M. (2010). Secretion of an endogenous subtilisin by *Pichia pastoris* strains GS115 and KM71. Appl. Environ. Microbiol..

[19] Markošová K., Weignerová L., Rosenberg M., Křen V., Rebroš M. (2015). Upscale of recombinant α-L-rhamnosidase production by *Pichia pastoris* MutS strain. Front. Microbiol..

[20] Bojarová P., Kulik N., Slámová K., Hubálek M., Kotik M., Cvačka J., Pelantová H., Křen V. (2019). Selective β-*N*-acetylhexosaminidase from *Aspergillus versicolor*—A tool for producing bioactive carbohydrates. Appl. Microbiol. Biotechnol..

[21] Weignerová L., Marhol P., Gerstorferová D., Křen V. (2012). Preparatory production of quercetin-3-β-D-glucopyranoside using alkali-tolerant thermostable α-L-rhamnosidase from *Aspergillus terreus*. Bioresour. Technol..

[22] Faury G., Molinari J., Rusova E., Mariko B., Raveaud S., Huber P., Velebný V., Robert A.M., Robert L. (2011). Receptors and aging: Structural selectivity of the rhamnose-receptor on fibroblasts as shown by Ca^2+^-mobilization and gene-expression profiles. Arch. Gerontol. Geriatr..

[23] Vishniac W., Santer M. (1957). The thiobacilli. Bacteriol. Rev..

[24] Bassanini I., Krejzová J., Panzeri W., Křen V., Monti D., Riva S. (2017). A sustainable one-pot two-enzyme synthesis of naturally occurring arylalkyl glucosides. ChemSusChem.

