# Supplementary material for: Bioproduction of Quercetin and Rutinose Catalyzed by Rutinosidase: Novel Concept of “Solid State Biocatalysis”
Source: Int J Mol Sci. 2019 Mar 5;20(5):1112. doi: 10.3390/ijms20051112 (PMC6429052; doi:10.3390/ijms20051112)
Supplement: Supplementary file 1 [file ijms-20-01112-s001.pdf]

Supplementary Material

Bioproduction of Quercetin and Rutinose Catalyzed by Rutinosidase: Novel Concept of "Solid State Biocatalysis".

Jana Kapešová[1], Lucie Petrásková[1], Kristína Markošová[2], Martin Rebroš[2], Michal Kotik[1], Pavla Bojarová[1], Vladimír Křen[1,*]

[1] Institute of Microbiology of the Czech Academy of Sciences, Laboratory of Biotransformation, Vídeňská 1083, CZ 14220, Prague 4, Czech Republic; hoficek@centrum.cz (J.K.); petraskova@biomed.cas.cz (L.P.); kotik@biomed.cas.cz (M.K.); bojarova@biomed.cas.cz (P.B.); kren@biomed.cas.cz (V.K.)

[2] Institute of Biotechnology, Slovak University of Technology, Radlinského 9, SK 81237, Bratislava, Slovakia; martin.rebros@stuba.sk (M.R.); kristina.markosova@stuba.sk (K.M.)

* Correspondence: kren@biomed.cas.cz; Tel.: +420-296-442-510 (V.K.)

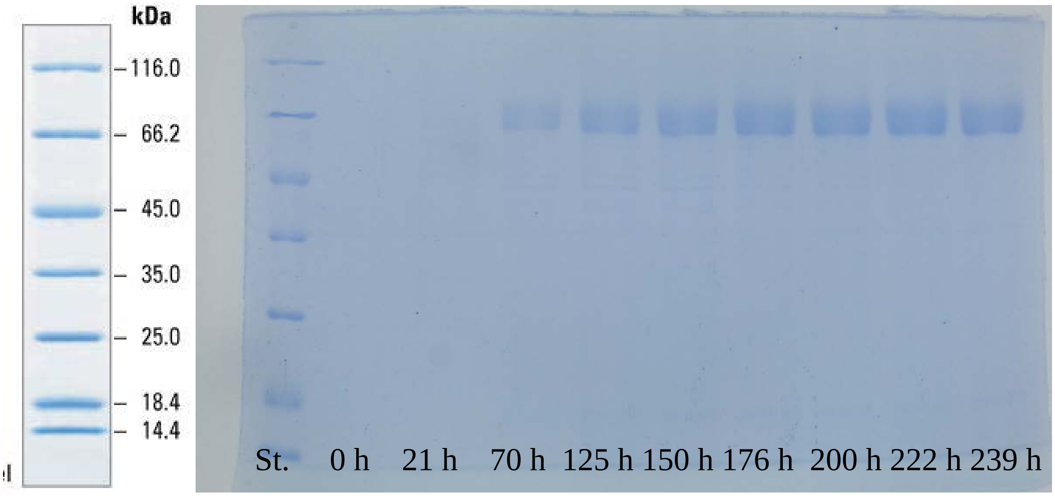

Fig. S1. SDS-PAGE electrophoresis of rutinosidase produced in the fed-batch fermenter; samples from various times of fermentation [hours].

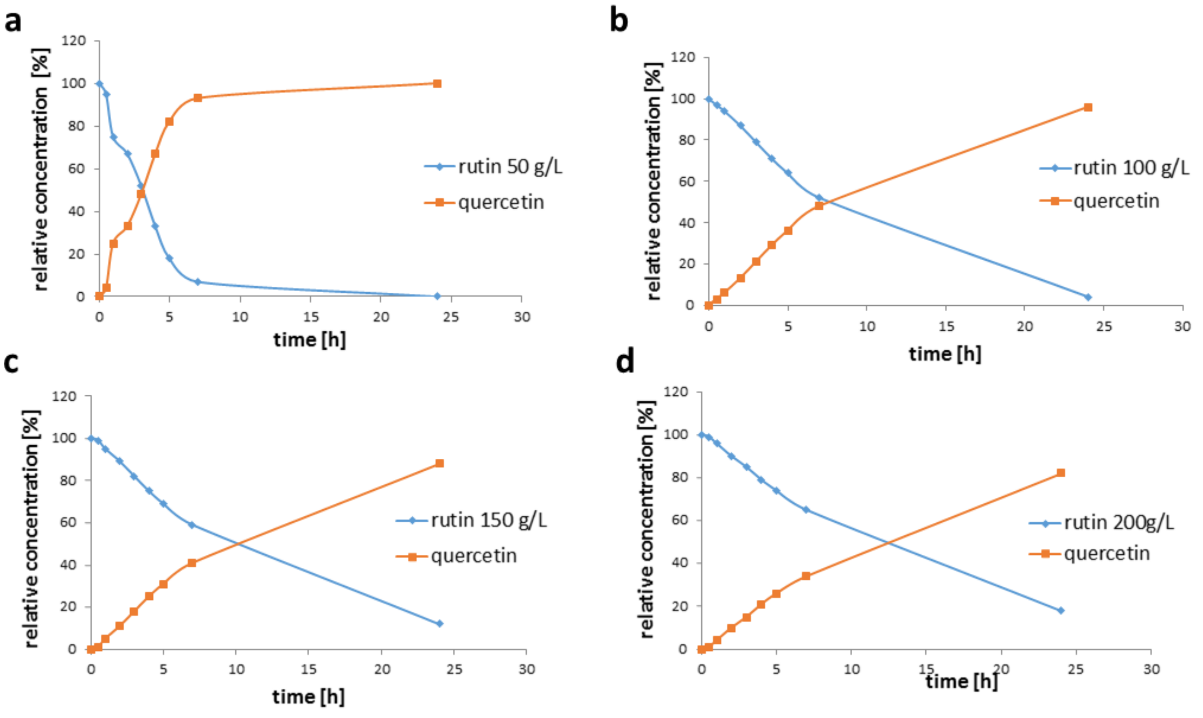

Fig. S2. Bioconversion of rutin to quercetin by crude wild-type rutinosidase. Reaction volume 40 mL; 40 °C; pH 3; rutinosidase activity 0.15 U/mL; rutin concentration (a) 50 g/L; (b) 100 g/L; (c) 150 g/L; (d) 200 g/L.

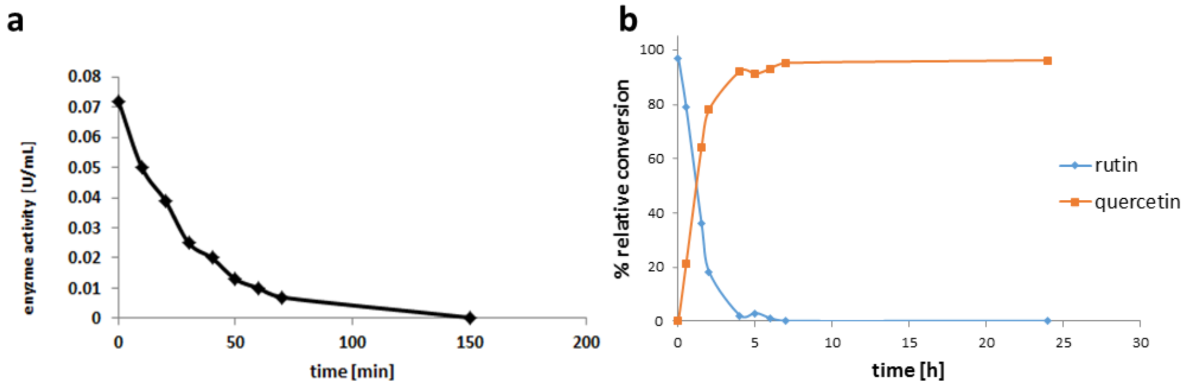

Fig. S3. Performance of crude recombinant rutinosidase at 50 °C. (a), Enzyme stability at 50 °C (crude medium, pH 3.0, flask fermentation, dialyzed in cellulose tubing cut-off 10 kDa against 10 mM sodium acetate buffer pH 3.6 for 2 h). (b), Bioconversion of rutin to quercetin at 50 °C (0.16 U/mL, dialyzed crude medium, reaction volume 3 mL, pH 3.0, 200 g rutin/L).

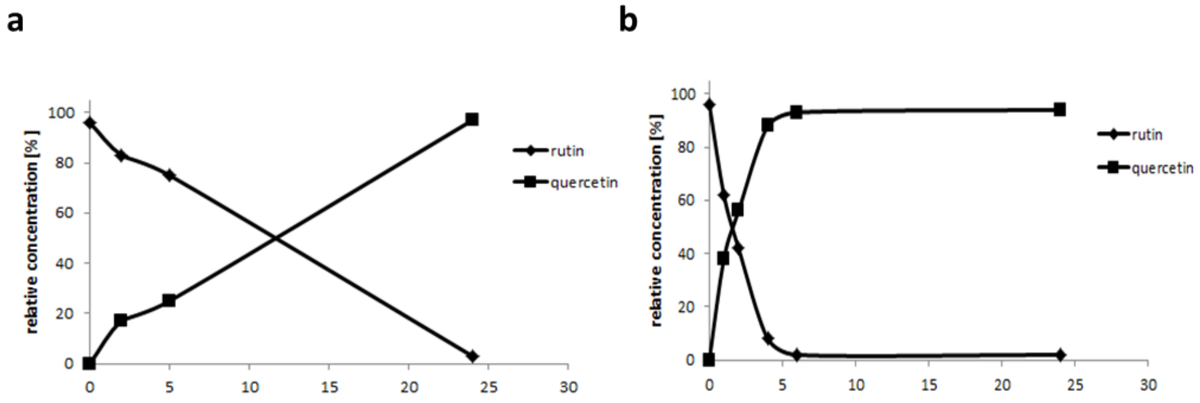

Fig. S4. Bioconversion of rutin with lyophilized crude recombinant rutinosidase resuspended in 50 mM citrate-phosphate buffer, pH 3.0 (a) after slow freezing and 3-month storage at −20 °C or (b) after shock freezing. Reaction conditions: rutin concentration 200 g/L; reaction volume 3 mL; 40 °C; pH 3.0; amount of rutinosidase 0.2 U/mL.

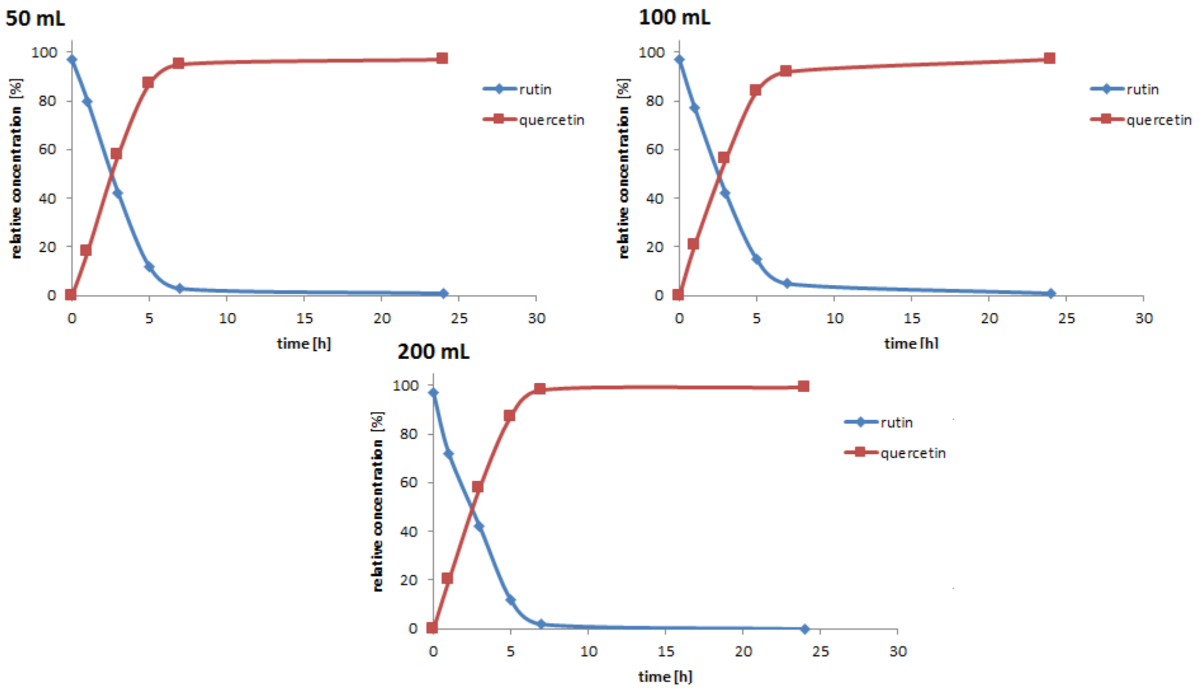

Fig. S5. Scale-up of rutin bioconversion under optimum conditions. Rutin concentration 200 g/L; reaction volume 50 mL; 100 mL or 250 mL; 40 °C; pH 3; crude medium from fermenter with rutinosidase activity 0.2 U/mL.
